# Loss of aquaporin 3 protein expression constitutes an independent prognostic factor for progression-free survival: an immunohistochemical study on stage pT1 urothelial bladder cancer

**DOI:** 10.1186/1471-2407-12-459

**Published:** 2012-10-08

**Authors:** Wolfgang Otto, Peter C Rubenwolf, Maximilian Burger, Hans-Martin Fritsche, Wolfgang Rößler, Matthias May, Arndt Hartmann, Ferdinand Hofstädter, Wolf F Wieland, Stefan Denzinger

**Affiliations:** 1 St. Josef Medical Centre, Department of Urology of Regensburg University, Regensburg, Germany; 2 Mainz University Medical Center, Johannes Gutenberg University, Department of Urology, Mainz, Germany; 3 Department of Urology, University of Würzburg, Würzburg, Germany; 4 Department of Urology, Klinikum St. Elisabeth, Straubing, Germany; 5 Institute of Pathology, University of Erlangen, Erlangen, Germany; 6 Institute of Pathology, University of Regensburg, Regensburg, Germany

**Keywords:** Urothelial bladder carcinoma, Stage pT1, Aquaporin 3 protein, Immunohistochemistry, Progression

## Abstract

**Background:**

Treatment of patients with stage pT1 urothelial bladder cancer (UBC) continues to be a challenge due to its unpredictable clinical course. Reliable molecular markers that help to determine appropriate individual treatment are still lacking. Loss of aquaporin (AQP) 3 protein expression has previously been shown in muscle-invasive UBC. The aim of the present study was to investigate the prognostic value of AQP3 protein expression with regard to the prognosis of stage pT1 UBC.

**Method:**

AQP 3 protein expression was investigated by immunohistochemistry in specimens of 87 stage T1 UBC patients, who were diagnosed by transurethral resection of the bladder (TURB) and subsequent second resection at a high-volume urological centre between 2002 and 2009. Patients underwent adjuvant instillation therapy with Bacillus Calmette-Guérin (BCG). Loss of AQP3 protein expression was defined as complete absence of the protein within the whole tumour. Expression status was correlated retrospectively with clinicopathological and follow-up data (median: 31 months). Multivariate Cox regression analysis was used to assess the value of AQP3 tumour expression with regard to recurrence-free (RFS), progression-free (PFS) and cancer-specific survival (CSS). RFS, PFS and CSS were calculated by Kaplan-Meier analysis and Log rank test.

**Results:**

59% of patients were shown to exhibit AQP3-positive tumours, whereas 41% of tumours did not express the marker. Loss of AQP3 protein expression was associated with a statistically significantly worse PFS (20% vs. 72%, p=0.020). This finding was confirmed by multivariate Cox regression analysis (HR 7.58, CI 1.29 – 44.68; p=0.025).

**Conclusions:**

Loss of AQP3 protein expression in pT1 UBC appears to play a key role in disease progression and is associated with worse PFS. Considering its potential prognostic value, assessment of AQP3 protein expression could be used to help stratify the behavior of patients with pT1 UBC.

## Background

Being a matter of debate for more than 100 years, the molecular basis of water transport across epithelial surfaces was first described in red blood cells in the late 1980s by Peter Agre and associates [1]. Later on, water-transporting channels were also shown to be present in renal epithelial cells and subsequently termed aquaporins (AQP) [2-4]. AQPs are a family of transmembrane proteins that selectively allow water or water plus other small, uncharged molecules such as urea and glycerol to pass along hydrostatic and osmotic gradients. Aquaporins are ubiquitously expressed in bacterial, animal and human cells. Hence, they are essential for cellular function [5]. To date, 13 different mammalian AQPs have been identified at the molecular level and localised to particular tissues [6]. Analysis of several human diseases has confirmed that AQPs are involved in various pathological conditions and provide promising drug targets [7,8]. Moreover, there is strong presumptive evidence that AQPs play a role in carcinogenesis, specifically in tumour angiogenesis and cell migration [9]. The pro-tumourigenic effect of a lost AQP expression in neoplastic cells has been the subject of previous studies. Knockdown of AQP3 for instance has been shown to be associated with increased migration and proliferation of gastric cancer cells [10].

There is only very limited data on expression and biological significance of aquaporins in human urothelium. Rubenwolf et al. were the first to characterize human urothelium with regard to all 13 members of the human AQP family. While transcripts for AQP3, AQP4, AQP7, AQP9 and AQP11 were detected in freshly-isolated urothelium and cultured urothelial cells by reverse transcriptase-polymerase chain reaction (RT-PCR), AQP3 was unequivocally expressed also at the protein level, with intense immunohistochemical labelling of the cell membranes of basal and intermediate layers in normal bladder urothelium [11].

To date, investigations into the potential significance of aquaporins in urothelial bladder carcinoma (UBC) are lacking [12]. In a preliminary analysis of AQP3 expression in UBC of various stages, our group demonstrated loss of AQP3 in muscle-invasive disease whereas stage Ta specimens were shown to invariably express the marker. Interestingly, 60% of pT1 tumours were found to be AQP3 positive while the remaining specimens revealed complete absence of AQP3. This finding suggests that AQP3 could be of value as a prognostic marker, particularly in the highly heterogeneous subgroup of pT1 patients [13]. The objective of the present study was to analyze the prognostic value of AQP3 protein expression in stage pT1 UBC patients. Despite numerous previous attempts to identify clinical and histopathological prognostic parameters, Shahin’s rule of each 30% of patients developing either never recurrence, recurring or even dying of the disease is still valid [14].

## Methods

### Patient characteristics

Tumour specimens of 98 patients, who were diagnosed between 2002 and 2009 with pT1 UBC by transurethral resection of the bladder (TURB) and second resection after 4–6 weeks were included in the present study. After second resection (re-resection) all patients received adjuvant instillation therapy with Bacillus Calmette-Guérin (BCG). Progression to muscle-invasive disease resulted in CX. TURB and all further treatment was carried out at a single tertiary high-volume referral center according to the European Association of Urology guidelines. Collection of tissue specimens had the approval of the local research ethics committee and full informed patient consent.

We retrospectively reviewed the histopathological and clinical data of all patients in relation to tumour recurrence, disease progression, and cancer-specific survival. Median follow-up was 31 months (range: 5–85 months).

### Histopathological assessment

All bladder tumours were evaluated using the TNM classification [15] and given recent findings that the WHO classification of 1973 is superior to later versions in terms of prognosis of non muscle-invasive bladder cancer patients, we used the WHO 1973 classification for further analysis [16,17]. Histopathological assessment was performed by two expert uropathologists (A.H., F.H.).

### Immunohistochemical assessment and analysis

Surgical samples were fixed in 10% formalin, dehydrated and embedded in paraffin wax. Dewaxed 4μm tissue sections were subjected to antigen retrieval by boiling for 10 min in tris-ethylenediaminetetraacetic acid (Tris-EDTA; pH 9) before labelling with titrated primary antibody (polyclonal anti-AQP3, host: rabbit, antigen: human AQP3, dilution 1:2000, Abcam, USA) for 16h at 4°C. Sections of normal urothelium within the TURB specimen served as internal control. In additon, positive control tissues of normal human urothelium known to express the antigen and negative controls in which the primary antibody was omitted were included in all experiments to ensure the immunohistochemistry was performed correctly.

**Figure 1:**
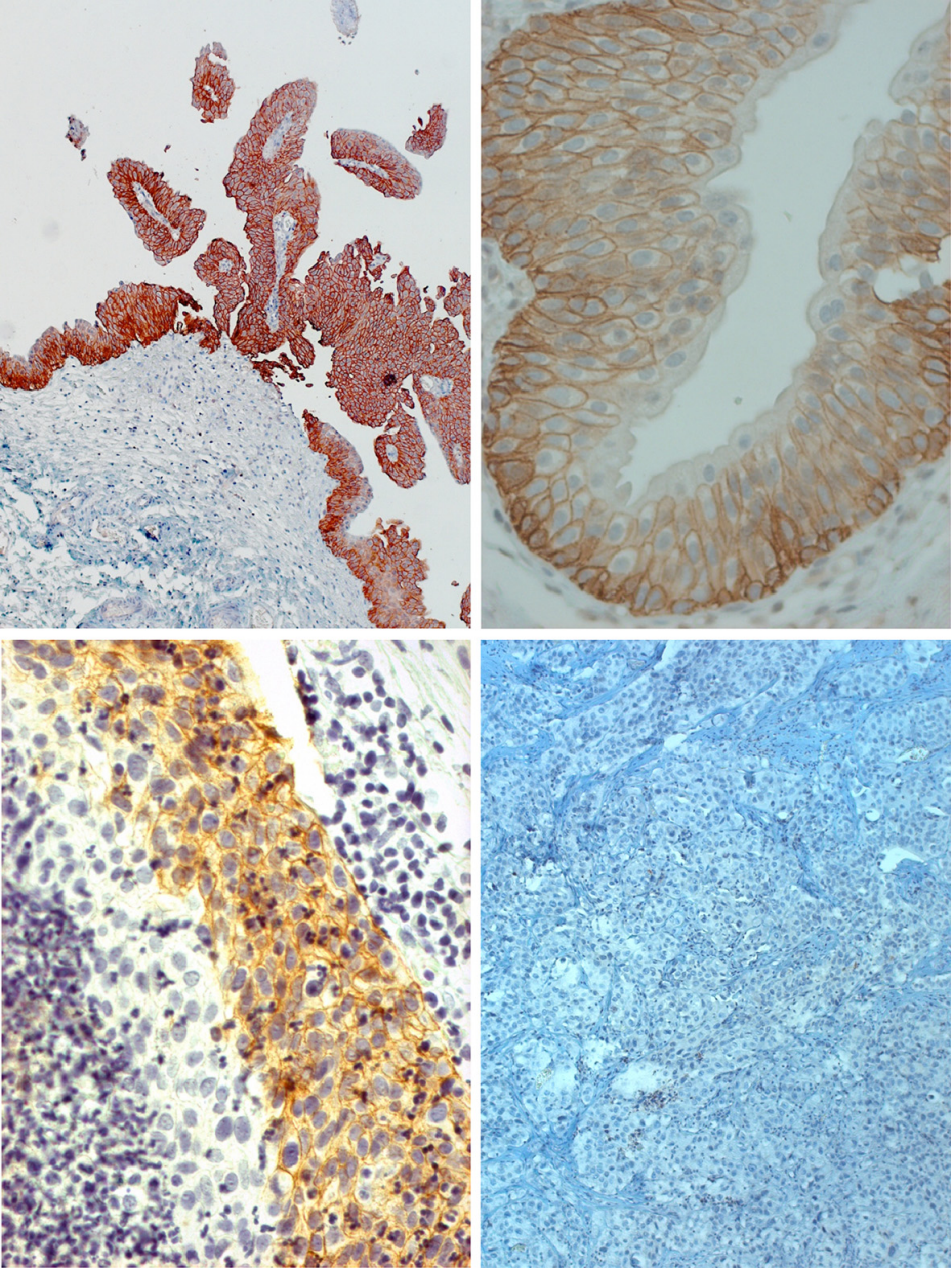
**Examples of various patterns of AQP3.** Homogeneous, cell membrane-associated expression of AQP3 in a low-grade papillary tumour (top left, 10-fold magnification). Regular expression in the basal and suprabasal, but not superficial cell layers in a section of normal human ureter (top right, 40-fold magnification). Heterogeneous expression of AQP3 in a high-grade pT1 tumour (bottom left, 40-fold magnification). Lack of expression in another pT1G3 UBC specimen (bottom right, 10-fold magnification).

Evaluation of the slides was performed without knowledge of patient-related data exactly as previously described [11,13]. Expression of AQP3 was visualised on a Primo Star microscope (Carl Zeiss Microimaging, Jena, Germany) under 4, 20, 40 and 100-fold magnification (Figure 1). Tumours were classified as AQP3-negative only in case of complete lack of immunoreactivity within the complete tumour and simultaneous unequivocal expression of AQP3 by the positive control included in each sample. Accordingly, partial expression, i.e. a tumour exhibiting even small AQP3-immunoreactive areas, was classified as AQP3 positive. Tumour specimens of a total of 87 patients were eligible for immunohistochemistry. The remaining 11 patients were excluded from the analysis due to insufficient amount of tumour tissue (n=4), technical reasons (n=7), or loss of follow-up.

### Statistical analysis

SPSS version 19.0 (SPSS Inc., Chicago, IL, USA) was used for statistical analysis. We compared different patient characteristics and clinicopathological parameters in relation to the aquaporin 3 protein expression status of the study patients by Fisher’s exact test. Multivariate Cox regression analysis was used to demonstrate the potential clinical value of AQP3 expression and other clinicopathological parameters for recurrence, progression and cancer-specific survival. Recurrence-free, progression-free and cancer-specific survival rates were calculated by Kaplan-Meier analysis and Log rank test. Values <0.05 were considered statistically significant.

## Results

### Histopathological assessment

The median age of the 87 patients was 70 years (range 41–98 years, 83% male). 86% of patients presented with an initial diagnosis of stage pT1 UBC. Patient-related and histopathological data are summarized in Table 1. There was no statistically significant difference with regard to clinical and pathological parameters between AQP3+ and AQP3- tumours.

**Table 1:** Patient charcateristics and clinicopathological parameters in relation to aquaporin 3 protein expression status

**Parameter**	**Total (%)**	**AQP3 positive (%)**	**AQP3 negative (%)**	**p value**
*Number of patients*	87 (100.0)	51 (58.6)	36 (41.4)	
*Gender*				
Female	15 (17.2)	7 (46.7)	8 (53.3)	p=0.227
Male	72 (82.8)	44 (61.1)	28 (38.9)	
*Age*				
median age (years)	70 ±11.5	68 ±12.6	73 ±9.3	p=0.095
age range (years)	41-98	41-98	50-88	
*History*				
first diagnosis of UBC	75 (82.8)	43 (57.3)	32 (42.7)	p=0.390
recurrent tumour	12 (17.2)	8 (66.7)	4 (33.3)	
*WHO grading 1973*				
pT1G2	15 (17.2)	8 (53.3)	7 (46.7)	p=0.429
pT1G3	72 (82.7)	43 (59.7)	29 (40.3)	
*Cofactors*				
associated CIS	36 (41.4)	20 (55.6)	16 (44.4)	p=0.394
multifocal tumours	17 (19.5)	11 (64.7)	6 (35.3)	p=0.388
tumour size ≥3cm	41 (47.1)	27 (65.9)	14 (34.1)	p=0.141
*BCG instillations*				
≤ 6 instillations	68 (78.2)	42 (61.8)	26 (38.2)	p=0.194
> 6 instillations	19 (21.8)	9 (47.4)	10 (52.6)	

*AQP3* aquaporin 3 protein; *BCG* Bacillus Calmette-Guérin; *CIS* carcinoma in situ; *UBC* urothelial bladder carcinoma; *WHO* world health organisation.

### Subsequent treatment and clinical course of patients

Following diagnosis of stage T1 UBC patients underwent instillation therapy with BCG with a median number of 6 instillations (range: 5–12 instillations). A total of 12 instillations, as recommended by the EAU guidelines, were completed by 19% of patients only due to irritative symptoms or low patient’s compliance. 23% of the patients had tumour recurrence, half of whom (55%) developed tumour progression (stage ≥pT2). All patients with recurrent disease underwent cystectomy. All patients who developed muscle-invasive disease were excluded from the study.

### Expression of AQP3 in relation to clinicopathological parameters

59% of patients were shown to exhibit AQP3-positive tumours, whereas 41% of patients and tumours, respectively, did not express the marker. Marker expression was independent of age, gender, patient history regarding UBC, WHO grading and histopathological cofactors such as carcinoma in situ (CIS), multifocal tumours and tumour size ≥3cm as well as the number of BGC instillations (see Table 1).

### Kaplan-Meier analysis of AQP3 expression in relation to prognosis

**Figure 2:**
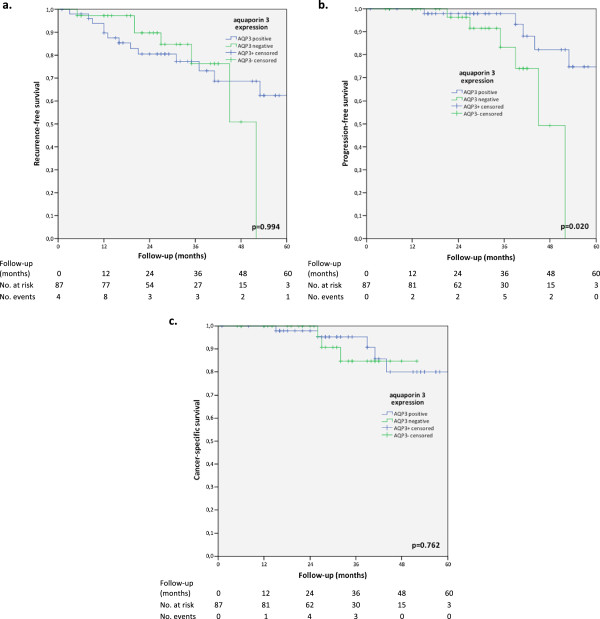
**Kaplan-Meier analysis of recurrence-free (RFS) (a), progression-free (PFS) (b) and cancer-specific survival (CSS) (c) in relation to aguaporin 3 expression in stage T1 urothelial bladder carcinoma.** While the lower RFS of AQP3 negative patients did not reach statistical significance, loss of AQP3 was associated with a statistically significantly worse PFS. There was no statistically significant difference in outcome regarding CSS.

1-, 2- and 4-year recurrence-free survival (RFS) was 90%, 79% and 22% in patients exhibting AQP3-negative tumours compared to 80%, 77% and 60% in patients with AQP3-positive tumours. This difference was not statistically significant (p=0.994). 1-, 2- and 4-year progression-free survival (PFS) was 97%, 86% and 20% in patients with AQP 3-negative tumours and statistically significantly worse compared to 98%, 98% and 72% in patients exhibiting AQP3+ tumours (p=0.02). By contrast, 1-, 2- and 4-year cancer-specific survival (CSS) showed no differences between AQP3+ (98%, 94% and 79%) and AQP3- (100%, 83%, 83%) tumour status (p=0.762). Kaplan-Meier curves of RFS, PFS and CSS in relation to AQP3 expression is illustrated in Figure 2.

### Multivariate Cox regression analysis of clinicopathological parameters and AQP3 expression in relation to prognosis

Multivariate Cox regression analyses for RFS, PFS and CSS revealed statistically significant differences between AQP3 positive and negative tumours in relation to PFS. Loss of AQP3 was independently associated with a worse PFS (HR 7.58, CI 1.29 – 44.68; p=0.025). Results are shown in Table 2.

**Table 2:** Multivariate Cox regression analysis of clinicopathological parameters and aquaporin 3 protein expression by the tumour regarding recurrence free (a), progression-free (b) and cancer-specific survival (c) in stage T1 urothelial bladder carcinoma

	**HR (CI 95%)**	**p-value**
**a.**		
**Gender***female vs. male*	0.50 (0.11 – 2.29)	0.372
**Age***per year*	1.01 (0.97 – 1.05)	0.770
**Recurrent disease***yes vs. no (initial finding)*	0.58 (0.10 – 3.50)	0.552
**Grading WHO 1973***G3 vs. G2*	1.08 (0.25 – 4.67)	0.916
**Associated CIS***yes vs. no*	1.58 (0.63 – 3.97)	0.328
**Focality***multifocal vs. unifocal*	0.33 (0.07 – 1.65)	0.177
**Tumour size***≥3cm vs. <3cm*	0.68 (0.25 – 1.92)	0.485
**BCG instillations***≤6 vs. >6*	0.77 (0.23 – 2.57)	0.667
**AQP3***negative vs. positive expression*	0.81 (0.29 – 2.25)	0.552
**b.**		
**Gender***female vs. male*	0.42 (0.03 – 5.27)	0.500
**Age***per year*	1.01 (0.95 – 1.08)	0.697
**Recurrent disease***yes vs. no (initial finding)*	4.08 (0.24 – 69.74)	0.332
**Grading WHO 1973***G3 vs. G2*	0.71 (0.28 – 1.79)	0.469
**Associated CIS***yes vs. no*	1.40 (0.28 – 7.12)	0.683
**Focality***multifocal vs. unifocal*	constant	0.976
**Tumour size***≥3cm vs. <3cm*	2.64 (0.30 – 22.97)	0.380
**BCG instillations***≤6 vs. >6*	constant	0.977
**AQP3***negative vs. positive expression*	**7.58 (1.29 – 44.68)**	**0.025**
**c.**		
**Gender***female vs. male*	constant	0.972
**Age***per year*	1.03 (0.97 – 1.09)	0.338
**Recurrent disease***yes vs. no (initial finding)*	constant	0.999
**Grading WHO 1973***G3 vs. G2*	constant	0.975
**Associated CIS***yes vs. no*	0.79 (0.18 – 3.46)	0.756
**Focality***multifocal vs. unifocal*	constant	0.976
**Tumour size***≥3cm vs. <3cm*	2.98 (0.35 – 24.86)	0.316
**BCG instillations***≤6 vs. >6*	constant	
**AQP3***negative vs. positive expression*	1.16 (0.26 – 5.15)	0.849

Reference in italics; p-values <0.05 are indicated in bold; *AQP3* aquaporin 3 protein; *BCG* Bacillus Calmette-Guérin; *CI* confidence interval; *CIS* carcinoma in situ; *HR* hazard ratio; *WHO* world health organisation.

## Discussion

Stage pT1 urothelial bladder carcinoma continues to be a challenging tumour entity for urologists due to unpredictable clinical courses. While about one third of patients never experience tumour recurrence, at least one third requires radical cystectomy for progression to muscle-invasive disease and the remaining 30% of patients ultimately die of UBC [14].

Numerous research groups worldwide have scrutinized a wide array of parameters to identify prognostic factors that may help to predict tumour progression in stage pT1 UBC [18-24]. Among these parameters grading appears to constitute the most powerful prognostic histopathological feature. In their analysis of a large collective of 310 stage pT1 UBC patients, Otto et al. only recently were able to show that tumour grading, specifically the distinction between grade 2 and 3 tumours as classified by the WHO classification of 1973, was the most consistent and reliable predictor in respect to prognosis [16]. Moreover, a series of immunohistochemical investigations into the prognostic value of various proteins expressed by UBC was performed, most of which provided disappointing findings. In a cohort of 175 patients, 19% of whom diagnosed with pT1G3 UBC, Rodriguez Alonso and associates identified over-expression of tumour suppressor protein p53 by the tumour as prognostic factor. Overexpression of p53 and ki67 alongside CIS, multifocality and solid bladder tumours were associated with a worse progression-free survival in multivariate analysis [25]. Lopez-Beltran et al. were able to demonstrate a prognostic value of different biomarkers in relation to patient survival. In a total of 51 patients consisting of pT1G3 tumours, expression of p53 alongside other cell cycle regulators such as cyclin D1 and cyclin D3 showed prognostic significance for PFS in multivariate analysis [26]. By contrast, Park et al. were unable to find any prognostic value for seven markers including p53 and ki67 in 61 patients with pT1G3 UBC [27].

Our previous finding of AQP3 transcript and protein expression in normal human urothelium and cultured urothelial cells prompted us to investigate expression and potential significance in diseased urothelium including urothelial cancer [11,13]. In a preliminary analysis of AQP3 expression in tumour specimens of various stages of UBC, we demonstrated loss of AQP3 protein expression in muscle-invasive disease whereas pTa specimens were shown to invariably express the marker. It was of note that 40% of stage pT1 UBC tumours exhibited loss of AQP3 protein expression [13]. Hence, we concluded that AQP3 might constitute a prognostic marker for progression to muscle-invasive disease.

Our present data indicate a statistically significantly worse PFS in patients in whom immunohistochemistry revealed loss of AQP3 expression. This finding was confirmed by multivariate Cox regression analysis. By contrast, recurrent disease was independent of the AQP3 expression status. Taking both our present and previous findings into account, we hypothesize that expression of AQP3 in stage pT1 UBC is pathogenetically associated with non-muscle-invasive disease while loss of AQP3 may be part of a molecular program associated with progression to muscle-invasive UBC [13]. Moreover, abnormalities of chromosome 9p, where the AQP3 gene is located, are frequently present in UBC, adding further presumptive evidence for a role of AQP3. However, such hypotheses have to be addressed in further studies aimed at elucidating the biological significance of AQP3 expression in UBC.

The pro-tumourigenic effect of a loss of AQP3 has been investigated in previous studies on other tumour entities. Knockdown of AQP3 expression for instance has been shown to be associated with increased migration and proliferation of gastric cancer cell lines [12]. In contrast, Kuseyama et al. found a high level of expression of AQP3 in oral and esophageal squamous cell carcinomas (SCC) [28]. Xu and associates investigated the effect of AQP3 on matrix metalloproteinase in human gastric carcinoma cells and concluded that AQP3 may be a promising drug target [29]. In the field of urological neoplasms, Ismail et al. provided evidence that prostate cancer cell lines in vitro become more sensitive to cryotherapy after inhibiting AQP3 by mercuric chloride and AQP3 siRNA [30].

Although the present study did not investigate the underlying molecular mechanisms of AQP expression in urothelial carcinoma, our findings suggest that AQP3 may be an independent predictor of tumour progression from stage pT1 towards stage pT2 tumours. This finding may be of clinical value in that patients exhibiting loss of expression of AQP3 in their TURB specimens could benefit from aggressive surgical treatment in the form of early cystectomy. However, we do not attempt to over-interprete our findings. The retrospective design of the study and the distinction between AQP3-positive versus AQP3-negative tumours may be oversimplistic. A detailed analysis of the various expression patterns of AQP3 in tumours by scoring both the labeling intensity and the proportion of positively-labeled tumour areas were not performed due to the low number of AQP3-positive tumours (n=51). The lack of such subanalyses is undoubtedly a limitation of the present study. Future work analyzing an appropriate number of AQP3-positive tumours is required to address this important aspect. Moreover, investigations into the molecular mechanisms of AQP3 expression in urothelial carcinoma are required to understand its role in the pathogenesis of UBC.

## Conclusions

The present study is the first to investigate a larger patient cohort of pT1 urothelial bladder carcinomas for AQP3 protein expression. Lack of AQP3 protein expression in pT1 tumours was shown to be associated with progression towards muscle-invasive disease. By contrast, tumour recurrence was independent of AQP3 expression. Importantly, this association was independent of other clinicopathological parameters. We conclude that AQP3 expression could be used to help stratify the behavior of patients with pT1 UBC.

Our findings underline the importance of investigating expression and function of AQPs beyond water and solute transport in normal and neoplastic urothelium. Further studies into the molecular mechanisms of AQP3 expression as well as prospective multicentre studies are to be awaited before valid conclusions can be drawn from our present findings.

## Abbreviations

AQP: Aquaporin; AUC: Area under the curve; BCG: Bacillus Calmette-Guérin; CIS: Carcinoma in situ; CSS: Carcinoma-specific survival; HR: Hazard ratio; IHC: Immunohistochemistry; MMP: Matrix metalloproteinase; PFS: Progression-free survival; RFS: Recurrence-free survival; TURB: Transurethral resection of the bladder; UBC: Urothelial bladder carcinoma; WHO: World health organisation.

## Competing interests

The authors declare that they have no competing interests.

## Authors’ contributions

WO and PCR conceived the study, acquired the data and drafted the manuscript. They were equally involved in the analysis and interpretation of data. SD participated in the study’s design and draft of the manuscript. PCR, AH and FH performed histopathological and immunohistochemical assessment and evaluation of the specimens. WO and MM performed the statistical analysis. MB, HMF, WR and WFW participated in the study’s design and coordination and helped to draft the manuscript. All authors read and approved the final manuscript.

## Pre-publication history

The pre-publication history for this paper can be accessed here:

http://www.biomedcentral.com/1471-2407/12/459/prepub
